# Comparison of Kinematic Sequences During Curveball and Fastball Baseball Pitches

**DOI:** 10.3389/fspor.2021.699251

**Published:** 2021-09-09

**Authors:** Donna Moxley Scarborough, Pablo E. Colón, Shannon E. Linderman, Eric M. Berkson

**Affiliations:** Department of Orthopaedic Surgery, Massachusetts General Hospital and Harvard Medical School, Boston, MA, United States

**Keywords:** Kinematic Sequence, baseball, pitching, biomechanics, curveball, kinetic chain

## Abstract

Performance of a sequential proximal-to-distal transfer of segmental angular velocity (or Kinematic Sequence) is reported to reduce stress on musculoskeletal structures and thus the probability of injury while also maximizing ball velocity. However, there is limited investigation regarding the Kinematic Sequence of the five body segments (Pelvis, Trunk, Arm, Forearm, and Hand) among baseball pitchers. Some biomechanical and epidemiology studies have reported an association of the curveball with increased risk for elbow injury among youth pitchers. Kinematic Sequences with altered distal upper extremity (forearm and hand) sequences have been associated with greater elbow valgus and shoulder external rotation torques compared to other Kinematic Sequences. Identifying Kinematic Sequence patterns during curveball pitches may lead to improved understanding of injury susceptibility. This study investigated the Kinematic Sequence patterns (and their variability) during curveball pitching and compared them to the sequences identified during fastball pitches. Using 3D motion analyses, 14 baseball pitchers (four high school, eight college, and two professional) performed 5–6 curveball pitches and 12 pitchers also threw fastball pitches in a simulated bullpen session. Eleven different curveball Kinematic Sequences were identified and 8 fastball Kinematic Sequences. There was no significant variability in the number of Kinematic Sequences performed between the two pitch types, (*Z* = −0.431, *p* = 0.67). The median number of KSs performed by each group was 2.5. The most frequently used Kinematic Sequences for both pitch types were due to alteration in the sequence of the distal segments. The total percentage of Kinematic Sequences with altered distal segment sequencing for the curveball pitches was 49% and 43% for fastball pitches. Identifying the frequency of Kinematic Sequences with altered timing of hand and forearm peak velocities across pitch types may lead to a better understanding of the stresses that individual pitchers incur.

## Introduction

Baseball pitching coaches and biomechanists encourage their players to throw in a sequential pattern, generating velocity from movement of the lower body to the torso and then out to the throwing hand. This sequence idealizes the generation of peak velocity for connected body segments to occur in a proximal-to-distal pattern. This target Kinematic Sequence (KS) is the most efficient pattern of movement for throwing which starts with the proximal segment (pelvis), trunk, arm, forearm, and ends with the distal segment, the hand (Putnam, 1993; Cheetham et al., 2008). Performance of this efficient transfer of segmental angular velocity is reported to reduce stress on musculoskeletal structures and thus the probability of injury while also maximizing ball velocity (Putnam, 1993; Seroyer et al., 2010; Chalmers et al., 2017; Scarborough et al., 2020). Recent investigations on a sample of fastball pitches performed by a group of high school, collegiate, and professional pitchers revealed that there are at least 17 distinct observable KS patterns (Scarborough et al., 2021). Out of the 17 observed patterns, none displayed the most efficient proximal-to-distal KS desired. Some of the KS patterns increased torques at the shoulder and elbow. These studies evaluated only the fastball pitch, but baseball pitchers commonly perform multiple pitch types during a typical game or bullpen session. Therefore, evaluation of other pitch types is needed in order to gain a better understanding of the biomechanical stresses that pitchers incur due to variant KS patterns. The curveball is a common breaking ball pitch frequently used among high school and more experienced pitchers. However, there is limited detailed biomechanical exploration of this pitch type despite an asserted increased risk for injury among youth pitchers (Fleisig et al., 2006; Nissen et al., 2009). Determining what KS patterns are commonly performed during curveball pitches as well as the variability of the KS patterns may provide added insight as to injury vulnerability.

It is generally assumed that as age and experience increase, that there is a reduction in variability of pitching biomechanics and an increase in the efficiency of the kinematic sequence of pitching (Stodden et al., 2005; Keeley et al., 2008; Fleisig et al., 2009; Nissen et al., 2009; Camp et al., 2017). Youth and high school pitch instructors typically emphasize performance of consistent pitch mechanics (Fleisig et al., 2009). While consistency of general pitch kinematics in experienced pitchers has been described by some investigators, others have found that mechanics of the pelvis and trunk are variable among experienced pitchers (Stodden et al., 2005; Fleisig et al., 2009; Urbin et al., 2013). As described by Urbin et al., during the throwing motion, the trunk contributes the proximal positioning for the scapulae and humerus (Urbin et al., 2013). Therefore, the trunk assists in setting up the humeral positioning and subsequent distal segment orientation of the forearm and hand during pitching. This study reported that pitches with larger kinematic trunk motion compared to pitches with restricted trunk motion resulted in less overall movement pattern variability of the distal segments (Urbin et al., 2013). This report supports the idea that finding an efficient proximal segment movement pattern allows for less distal segment position variability. The kinematic variability described, likely influences segment angular velocity sequencing consequentially.

Influenced by the power and timing of trunk movement, torque developed during shoulder rotation is correlated to elbow valgus torque and subsequent injury risk (Chalmers et al., 2017; Aguinaldo and Escamilla, 2019). The proper execution of the proximal to distal kinematic sequence leads to less torque production across the shoulder and elbow yet contributes to increased ball speed (Putnam, 1993; Seroyer et al., 2010; Scarborough et al., 2021). Certain kinematic sequences result in greater shoulder and or elbow torques than other KS patterns (Aguinaldo and Escamilla, 2019; Scarborough et al., 2021). Specifically, torques increase among KS patterns where the trunk reaches peak rotational velocity prior to the peak pelvis velocity (Seroyer et al., 2010; Aguinaldo and Escamilla, 2020). This emphasizes a demand for deeper investigation of KS patterns across all pitch types and all levels of pitching.

This study aims to (1) Identify Kinematic Sequence patterns performed during the curveball pitch across pitchers of varied level of experience, (2) Determine the intra pitcher variability of the sequences performed by each pitcher during curveball pitches, and to (3) Compare the Kinematic Sequence patterns performed during the curveball and fastball pitches.

## Materials and Methods

### Participants

Fourteen baseball pitchers (mean age: 19.2 ± 2.9 years) underwent 3D biomechanical pitch analyses of 71 total curveball pitches. Four high school, eight collegiate, and two professional level baseball pitchers participated in this institutional review board approved cross sectional study, and all participants provided written informed consent prior to participation (Table 1). Each pitcher was required to participate on a competitive baseball team for at least 3 months per year and to be free of self-reported injury for at least 3 months prior to study enrollment. Exclusion criteria included a history of underlying neurological conditions that may influence upper extremity strength and movement patterns, or a known self-reported allergy to adhesives.

**Table 1:** Pitcher characteristics and average pitch speeds.

**Level**	**Height (m)**	**Weight (kg)**	**Age (year)**	**Curveball speed (m/s)**	**Fastball speed (m/s)**
High School	1.73	65.77	15	28.89	30.84
High School	1.75	70.31	14	27.29	32.84
High School	1.85	77.11	16	25.66	30.84
High School	1.91	96.36	18	26.55	31.62
Collegiate	1.70	65.91	18	26.48	32.67
Collegiate	1.78	72.57	19	28.59	33.26
Collegiate	1.78	77.27	22	32.3	34.03
Collegiate	1.80	86.36	22	27.89	32.32
Collegiate	1.88	90.91	22	28.23	34.41
Collegiate	1.91	86.36	19	31.32	37.88
Collegiate	1.91	100.00	20	29.33	33.69
Collegiate	1.96	86.36	18	29.81	33.07
Professional	1.85	102.06	23	30.68	37.60
Professional	1.88	102.06	23	32.04	38.39

### Data Collection Procedures

**Figure 1:**
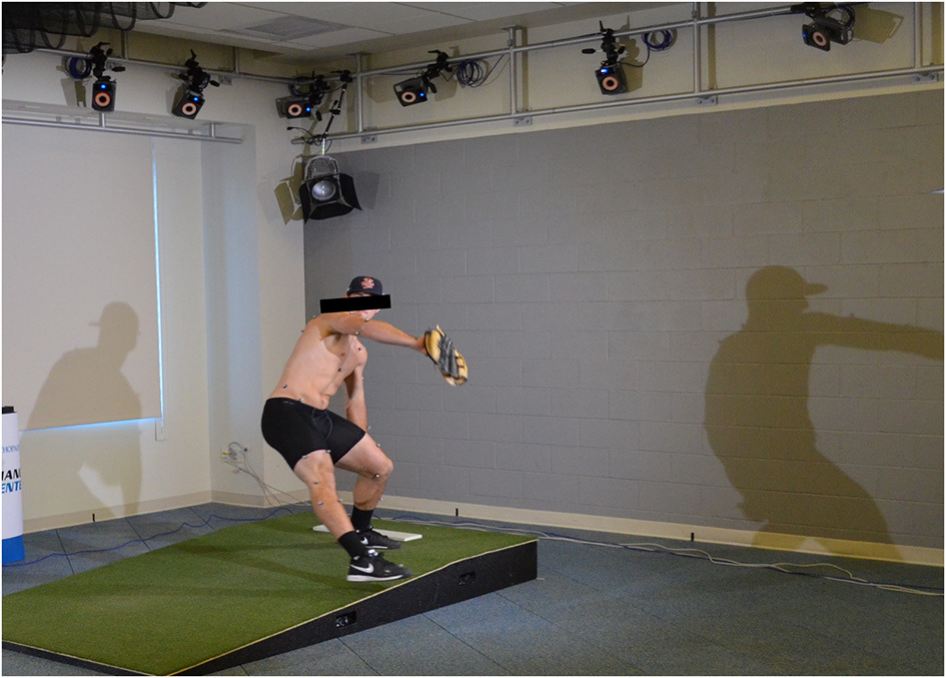
Illustration of collegiate baseball pitcher undergoing 3D motion capture.

Each participant underwent 3D motion capture analysis of their curveball pitching mechanics. An established motion capture marker set consisting of 62 (14 mm) reflective markers was applied to each pitcher with double sided toupee tape (Scarborough et al., 2020; Linderman et al., 2021). Markers were attached at specific anatomical locations in accordance with International Society of Biomechanics (ISB) recommendations for joint center and body segment axis definitions (Wu et al., 2002, 2005). Pitchers were instructed to perform their standard pre-bullpen warmup and stretching regimen, and were subsequently asked to throw a typical bullpen session including a minimum of 10 curveball pitches. During the bullpen session, 20 Vicon MX™ T-series cameras captured marker set positional data at 360Hz (Vicon Motion Systems Ltd., Oxford, Oxfordshire, UK) (Figure 1). Each pitch was thrown the standard home plate distance of 18.44 m from a turf mound to a target marked with a strike zone. Maximum pitch speed was also simultaneously collected using a radar gun (Stalker ATS 5.0 radar gun, Plano, TX, USA). The fastest and most accurate pitches based on strike zone impact location were selected for analyses.

Pitches that landed outside the established strike zone were excluded from analyses, as were any pitches where a motion capture marker became dislodged from a subject's body during pitching. Pitch velocity was captured in order to identify the mean pitch speed for all subjects in an effort to select the most representative and simultaneously on-target pitches for each subject in our cohort of pitchers competing at different levels of play. Utilizing these standards, five curveball pitches were analyzed for 13 pitchers and six curveball pitches were analyzed for one pitcher, for a total of 71 curveball pitches.

### Biomechanical Analysis

Data analysis was performed using Visual 3D™ biomechanics software (Version 5, C-Motion Research Biomechanics, Inc., Germantown, MD, USA). A fourth-order zero-lag Butterworth low-pass filter with a cut-off frequency of 18 Hz was used to process all marker position data.

Motion capture lab coordinates were defined for analyses with the *X*-axis in line with the subject's throwing direction, the *Z*-axis as the vertical direction, and the *Y*-axis as the cross-product of the *X* and *Z*-axes. Relative marker position data in 3D space was used to construct a 15-segment six degree-of-freedom (DoF) dynamic skeletal model of each subject.

All biomechanical variables of interest were derived from six DoF model calculations including:

Maximum angular velocity of the pelvis, trunk, arm, forearm, and hand segments. Body segments and local axes of rotation were based on ISB definitions and are listed in Table 2 (Wu et al., 2002, 2005). For the upper extremity, the wrist joint center was defined as the midpoint between markers placed on the radial and ulnar styloid processes, while the midpoint between markers placed on the medial and lateral humeral epicondyles defined the elbow joint. Markers placed on the dorsal acromioclavicular joint, trigonum spinae, inferior angle of the scapulae, angulus acromialis, coracoid process were used to define the shoulder girdle of the pitching arm. The forearm model segment was then established by the wrist and elbow joint centers, whereas the arm segment was defined by elbow and shoulder joint centers. Alignment of the *Z*-axis for the arm segment was established relative to the longitudinal axis of the humerus. Markers placed on bilateral anterior and posterior superior iliac spine pelvic landmarks defined the pelvis segment and a CODA pelvis model (Carnwood Dynamics, Ltd., Rothley, Leicester, UK) was employed (Hamill and Selbie, 2014). The angular velocity of each of the five body segments included in the kinematic sequence was calculated relative to the laboratory coordinate system.

**Table 2:** Anatomical locations of marker placements for kinematic sequence modeling.

**Marker placement**	**Body segment**
Third metacarpal	Hand
Middle of the dorsum of the forearm; Radial and ulnar styloid process (wrist); Medial and lateral humeral epicondyles (elbow)	Forearm
Middle dorsum of the upper arm; dorsal acromioclavicular joint, trigonum spinae, inferior angle of the scapulae, angulus acromialis, coracoid process (shoulder)	Arm
Sternum, clavicle, 7th cervical vertebrae, 10th thoracic vertebrae, middle of the right scapulae	Trunk
Anterior superior iliac spine posterior superior iliac spine, iliac crest	Pelvis

### Kinematic Sequence Definition

The angular velocity of individual modeled segments was calculated based on prior methodology used in KS investigations of throwing motions and golf swings (Putnam, 1993; Fortenbaugh et al., 2009). The total magnitude—the square root of the sum of the velocity in each plane (sagittal, frontal, and transverse) squared was calculated for each of the hand, arm, forearm, trunk, and pelvis segments.

Key time points the pitch cycle were identified including balance point (beginning of pitch, time point of first maximum knee elevation) and follow through (end of pitch, maximum shoulder internal rotation). The time point of maximum angular velocity for each of the aforementioned five model segments was calculated and rounded to the one ten-thousandth. The relative timing of the peak angular velocity for each segment was calculated relative to the start of the pitch (balance point). In this foundational kinematic sequence investigation of the curveball pitch, the relative timing of peak segmental angular velocities magnitudes was calculated for this timing-based classification study.

**Figure 2:**
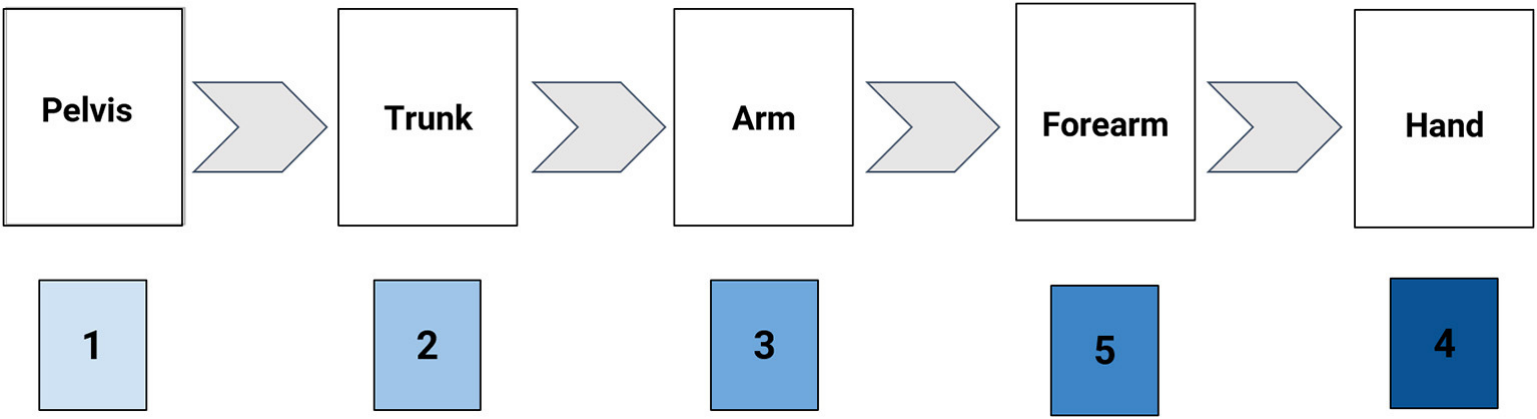
Illustration of the method of Kinematic Sequence naming convention (KS: 12354).

The kinematic sequence of each pitch was determined *via* the relative timing of the peak angular velocity for the five key body segments of interest based on prior work by Scarborough et al. (2020, 2021). Based on modeling efficiency during throwing, the ideal overhead pitch sequence is one where the timing of each body segment's peak angular velocity occurs in a proximal-to-distal (PDS) pattern (pelvis->trunk->arm->forearm-> hand) (Putnam, 1993). Therefore, kinematic sequences are named relative to this ideal proximal-to-distal segment pattern. A numerical placeholder is assigned to each body segment based on its ideal placement in a proximal-to-distal sequence (Scarborough et al., 2020). For example, peak pelvis angular velocity is expected to occur first, so it is assigned a value of “1.” Correspondingly, it is ideal for angular velocity of the hand to peak last so that maximum momentum is generated up through the lower and upper extremities and fully transmitted to the hand at pitching ball release. Therefore, the hand segment is designated with a “5” placeholder (Figure 2). The relative order of body segments' peak angular velocity occurring as part of the pitching motion sequence can thus be compiled and represented using this shorthand naming system.

The previously described Kinematics Sequence Classification groups the observed KSs to better understand the influences that altered sequences have on the pitcher's body. Each group is named relative to the first body segment that demonstrates peak angular velocity out-of-sequence from the targeted proximal-to-distal sequence (Scarborough et al., 2021). The groups described previously include: (1) the proximal-to-distal (PDS) group which also includes the closest to the ideal sequence where the forearm and the hand segments' peak angular velocities peak simultaneously, (2) the altered distal upper extremity (DUE) group, and (3) the altered proximal upper extremity (PUE) group and the altered pelvis/trunk (CORE) group (Scarborough et al., 2021).

### Data Analyses

Descriptive statistics were performed to meet the aims of the study. Kinematic Sequence variability within a pitcher was measured as the number of KSs performed for each pitch type. A non-parametric Wilcoxon signed-rank test was implemented to compare the number of kinematic sequences between fastball (FB) and curveball (CB) pitches among 12 pitchers who performed both pitch types. Two of the pitchers did not complete five fastball pitches and therefore were not included in this portion of the analyses. The IBM SPSS Statistics for Windows, version 24 statistical platform (IBM Corp., Armonk, NY) was used for statistical analyses. A *P*-value of <0.05 was set as the level of statistical significance.

## Results

### Kinematic Sequences Observed During the Curveball Pitch

Eleven different Kinematic Sequence patterns were identified across the 71 CB pitches (Table 3). None of the 71 pitches analyzed demonstrated the proximal-to-distal KS order (Kinematic Sequence 12345). The closest Kinematic Sequence to the target Proximal-to-Distal sequence was that of Pelvis->Trunk->Arm->simultaneous Forearm and Hand (Kinematic Sequence 12344).

**Table 3:** List of the Kinematic Sequences within each pitch type within the Kinematic Sequence Category groups.

**Sequence**	**PDS**	**CORE**	**PUE**	**DUE**
**Curveball**	12,344	11354	12443	12353
KS total = 11		21354	12453	12354
		21343	12543	
		21433	12533	
**Fastball**	12344	21354	12443	12353
KS total = 8			12453	12354
			12533	
			12543	

The most prevalent order for the CB pitches was pelvis-> trunk->arm->hand->forearm (Kinematic Sequence 12354). The two most prevalent KSs performed among the sample of CB pitches fell in the DUE sequence category and the third most prevalent category was in the PDS category (Table 4).

**Table 4:** The three most performed kinematic sequences in this curveball pitch data set.

**Sequence**	**1st** **segment**	**2nd** **segment**	**3rd** **segment**	**4th** **segment**	**5th** **segment**	***n***
12354 DUE	Pelvis	Trunk	Arm	Hand	Forearm	18
12353 DUE	Pelvis	Trunk	Arm^*^	Hand	Forearm^*^	17
12344 PDS	Pelvis	Trunk	Arm	Forearm^*^	Hand^*^	14

* *Indicates segments reaching simultaneous peak velocity*.

### Comparison of Intra-Pitcher Variability and Kinematic Sequences Performed Between the Curveball and Fastball Delivery

There was no significant difference between the number of KSs performed during the curveball and fastball across the 12 baseball pitchers (*Z* = −0.431, *p* = 0.67). The median number of KSs performed by each group was 2.5. The most frequently performed KSs for both curveball and fastball were within the DUE category.

The percent of observed Kinematic Sequences performed during the CB (44%) and FB (45%) pitches that demonstrated the altered distal upper extremity peal velocity sequence were very similar. Within the DUE Kinematic Sequence category, the percentage of KSs which follow the Pelvis->Trunk->Arm and hand simultaneously->forearm (KS 12353) for the CB pitches was 55% compared to 29% among the FB pitches (Tables 4, 5).

**Table 5:** The three most performed kinematic sequences in this fastball pitch data set.

**Sequence**	**1st** **segment**	**2nd** **segment**	**3rd** **segment**	**4th** **segment**	**5th** **segment**	***n***
12354 DUE	Pelvis	Trunk	Arm	Hand	Forearm	18
12453 PUE	Pelvis	Trunk	Forearm	Hand	Arm	13
12353 DUE	Pelvis	Trunk	Arm^*^	Hand	Forearm^*^	8

* *Indicates segments reaching simultaneous peak velocity*.

### Comparison of Intra-Pitcher Variability of Kinematic Sequence Patterns

**Figure 3:**
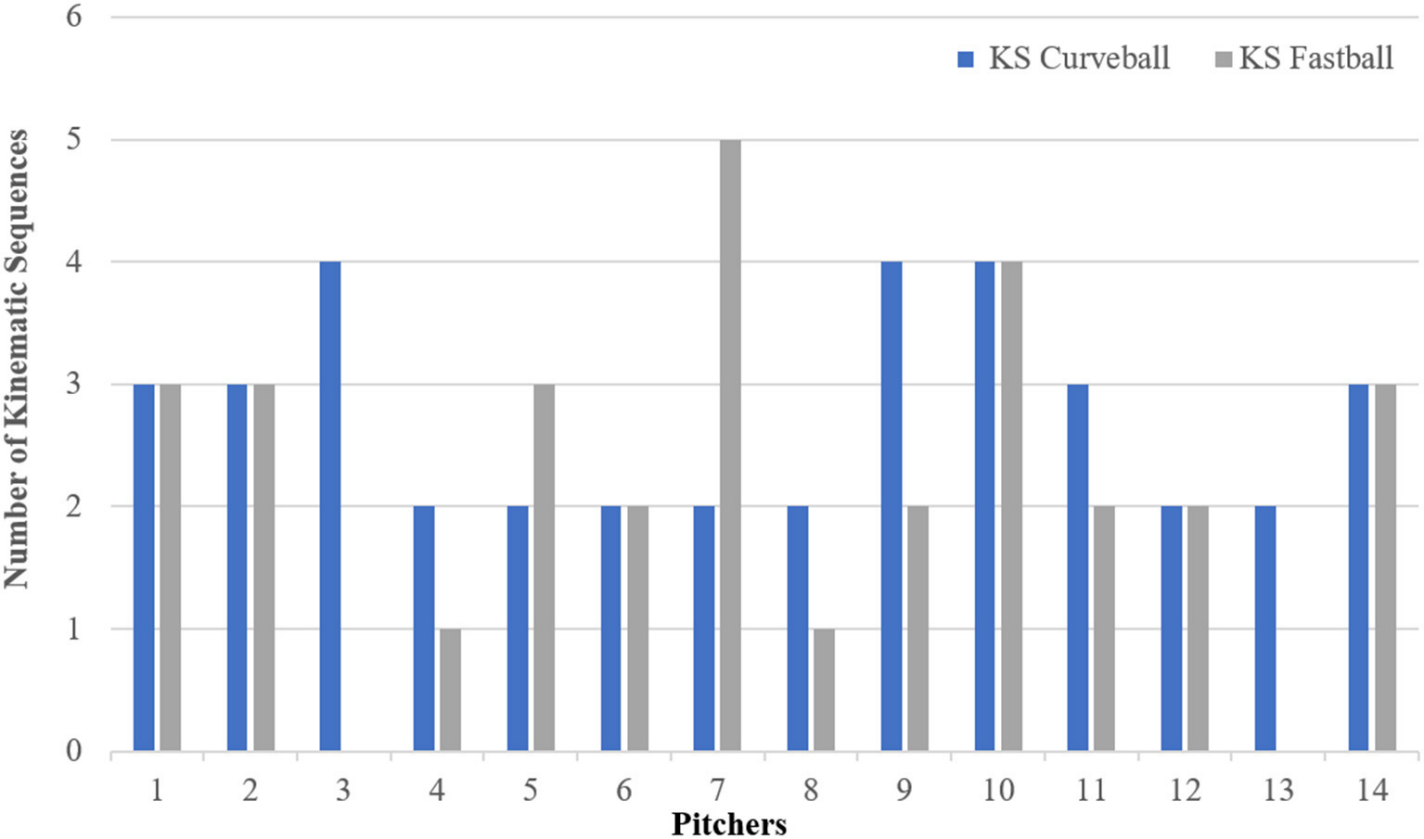
The number of Kinematic Sequences performed across the 14 pitchers for Curveball and Fastball pitches.

**Figure 4:**
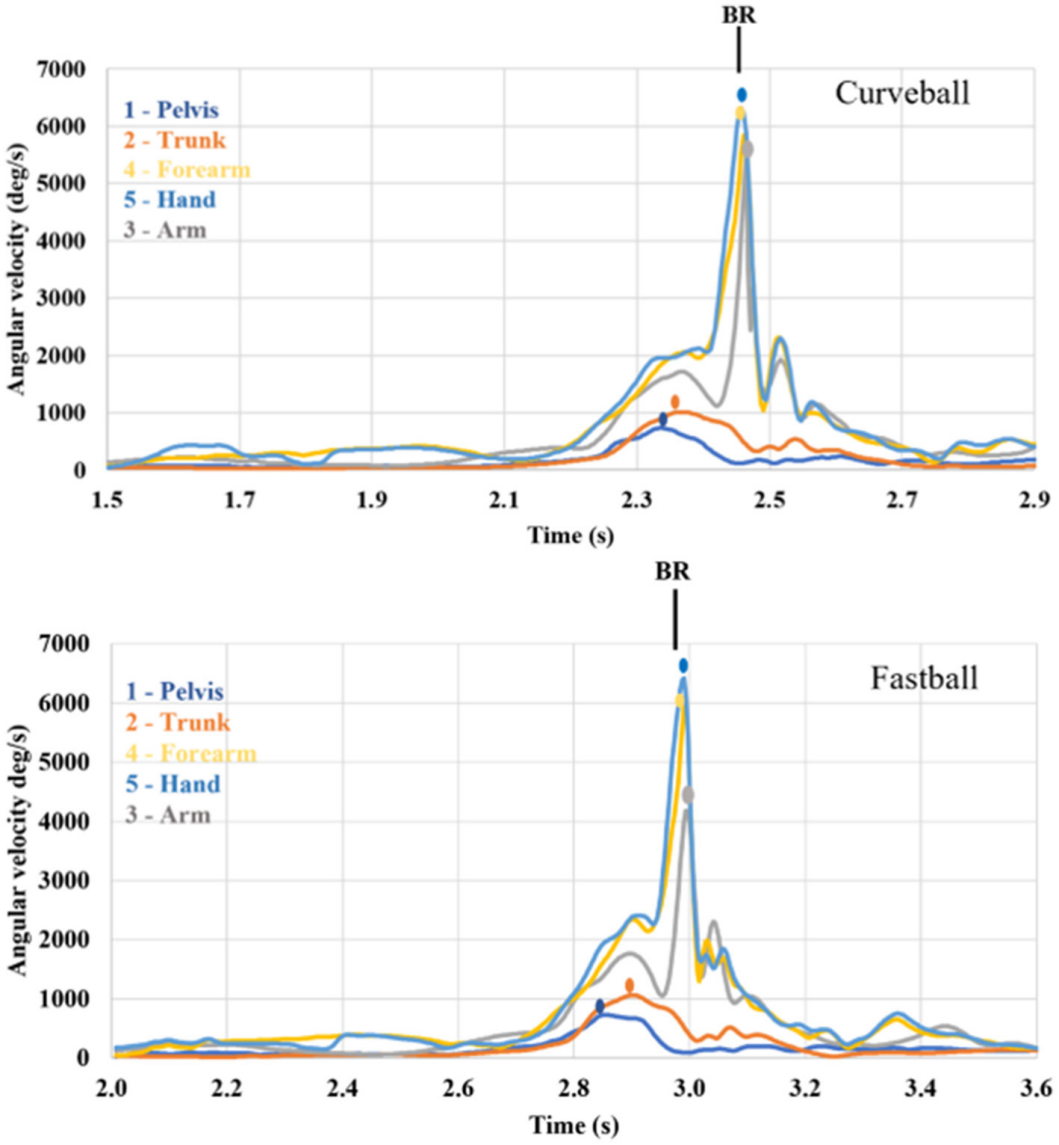
Kinematic sequence data from a pitcher at the professional level. Data from a single curveball and fastball are plotted starting at balance point (time of maximum leg lift—greatest hip flexion). The kinematic sequence pelvis -> trunk -> forearm -> hand -> arm (KS: 12453) demonstrates a proximal upper extremity altered sequence (PUE) and was performed during both pitch types. The black vertical line identifies time of ball release (BR) for each pitch example.

No players performed only one Kinematic Sequence. An average of 2.7 (SD = 0.83) different Kinematic Sequences were observed per pitcher across their 5–6 curveball pitches thrown (Figure 3). In Figure 4, data is presented as an example of a single pitcher performing the same KS for both CB and FB pitches.

## Discussion

This is the first study to identify and classify Kinematic Sequences of the curveball pitch and compare the findings with the KSs during the fastball pitch. In addition, this study investigates intra-pitcher variability of KSs during the curveball. Findings revealed that there are similarities in the number of KSs during both pitch types. However, some different KS patterns were observed between the fastball and curveball.

While neither the FB nor CB KSs demonstrated the ideal Kinematic Sequence patterns, they both demonstrate a predominance of the distal upper extremity category of Kinematic Sequence. The KS pattern where the shoulder and hand peaked simultaneously prior to the forearm occurred more frequently during the CB pitches than FB pitches. This KS sequence, recognized as 12353, has been reported in a previous study of FB pitches demonstrating a slightly lower average elbow valgus torque than the 12354 KS (Scarborough et al., 2021). It is unknown if similar findings of torque production occur in these curveball KS patterns. Future studies of torque production and differences between KS and joint stresses during the CB pitches are warranted.

Variability in the number of different Kinematic Sequence patterns that a pitcher performs during a set of one specific pitch type and then across different pitch types has been a recent focus in the pitching biomechanics literature. Several studies report that more experienced pitchers have less variability in temporal and kinetic pitching mechanics (Stodden et al., 2005; Urbin et al., 2013; Glanzer et al., 2019). However, others report that pelvis rotation velocity is variable across pitches (Fleisig et al., 2009). Because different KSs produce different stresses on the shoulder and elbow, one would expect less variability in higher level pitchers. A previous investigation, however, found that high school level pitchers demonstrated the least variability in the number of KSs performed during 10 FB pitches compared to more experienced pitchers (Scarborough et al., 2020). This finding promotes discussion around how performance of more than one KS pattern may benefit longevity in sport. Perhaps, avoidance of performing a single KS pattern associated with especially high upper extremity torque may offer protection against injury and allow for continued play with less injury risk. Our current study findings show that, like the variability of KSs during the FB, there is variability in the number of KSs during the curveball. Further investigation is required to characterize the strength of the relationship between variability in KSs patterns and long-term injury susceptibility.

Perfecting the biomechanics of the overhead throw is key towards minimizing stress and injury. Incidence of early trunk rotation has been associated with increased shoulder external rotation and increased valgus torque on the elbow (Oyama et al., 2014; Roach and Lieberman, 2014; Camp et al., 2017), and has also been more widely witnessed in younger pitchers (Aguinaldo and Escamilla, 2019). These findings suggest that inefficient KSs in younger pitchers are more likely to lead to injury. It is apparent, however, that coaches cannot possibly notice all variabilities in the sought-after proximal to distal Kinematic Sequence, as pitching motions are completed at such high speeds. In particular, it is more difficult to notice the differing peak moments across distal segments such as the shoulder, forearm, and hand. During the follow through of the CB pitch, peak moments of forearm supination—the movement that gives the CB its characteristic motion—can be hard to separate from the hand and shoulder (Grantham et al., 2015; Makhni et al., 2018). This aspect of the CB throw is a coaching point for all athletes and should help guide coaches and athletes to develop better practicing habits to address errors related to ineffective timing of distal segments. By examining the common variabilities in KS patterns during the CB, as this study has, the sports medicine community in coalition with further research, can focus on the reasons these patterns arise and develop ways to fix them on the field to minimize stress and vulnerability of injury.

There are a limited number of investigations of same pitcher variability (intra-pitcher variability) especially with regard to timing of movement, like the kinematic sequence patterns. One study demonstrated that skill acquisition is based on experience of different movement patterns to learn which is most effective for the individual (Langendorfer and Roberton, 2002). The idea of using different movement patterns for skill acquisition likely is not limited to young pitchers but also may be contributing to some of the variability of the KSs observed across the collegiate and professional pitchers. Our study reveals that most pitchers demonstrate some KS variability. This foundational observation allows us to ask future questions about how many KSs is helpful to a pitcher for adaptation during a game, which KS patterns place too much stress about individual's shoulder or elbow and if variation in KS offers protection to such stresses of one particular Kinematic Sequence.

One of the underlying limitations in motion capture analysis is that despite using a camera system at one of the higher reported capture rates (360 Hz), the underlying question remains if results would vary with an even higher capture rate. Specifically, would the segments reported with simultaneous times of peak angular velocity actually fall into an order, and if so, what order? Use of faster camera technology in the future will hopefully elucidate this question. As with all laboratory-based studies, we also recognize that there are inherent limitations in extrapolation of pitch performance in controlled indoor lab settings to pitching on outdoor dirt mounds with added game day stresses. However, understanding the KSs performed in an environment without external factors is beneficial for first steps in studying variability in movement patterns. Variability of pitch biomechanics in published literature has been reported using samples of 6 to 15 pitches per player (Stodden et al., 2005; Urbin et al., 2013; Glanzer et al., 2019). A previous study reported variability of KSs on a sample of 10 pitches per player and found that one pitcher of the 22 studied performed 6 different fastball KSs. The average number of KSs performed by each pitcher was 3.2 distinct patterns (Scarborough et al., 2020). While we believe that this prior study finding justifies the use of a sample of 5 pitches per player for the investigation of variability in KSs. It is not known if for the CB pitch whether a larger sample size would change the average number of KSs performed. Future investigations looking at the variability of KSs as a pitcher reports fatigue or near the end of an outing may provide additional important information toward understanding KSs and the relationship to vulnerability of injury.

Although this study aimed to sample pitchers of different experience levels yielding 71 pitches, we recognize that this study is based upon a limited sample size of pitchers. However, this small sample size lays groundwork for understanding variability in CB kinematic sequences and understanding the potential risk for injury.

## Conclusions

This study of the CB pitches did not observe the sought after idealistic 5 segment proximal-to-distal pattern. Variability in the number of kinematic sequences was similar between the FB and CB pitches, and the most frequently performed Kinematic Sequence during the CB pitches consisted of forearm segment peak velocity after the peak velocity of the hand (KS 12354). The most frequently used Kinematic Sequences for both pitch types were due to alteration in the sequence of the distal segments. There were differences in which Kinematic Sequences were utilized between the FB and CB pitches. The total percentage of Kinematic Sequences with altered distal segment sequencing for the curveball pitches was 49% and 43% for fastball pitches. Since a proper execution of the Kinematic Sequence is thought to lead to decreased demand on shoulder and elbow structures while generating maximum ball speed (Seroyer et al., 2010), future investigation of Kinematic Sequence pattern analysis both across all common pitch types and all levels of competition is needed. Future analyses of mechanical load on the shoulder, elbow and wrist are warranted to better understand the physical repercussions that the different kinematic sequences have on joint health and longevity in sport. Knowing which sequences incur greater stresses could direct trainers to modify training regimes to better protect vulnerable anatomic structures. Similarly, pitch instructors could direct athletes to modify their biomechanics or the frequency of using the pitch types that are performed with Kinematic Sequences that produce greater mechanical joint loading.

## Data Availability Statement

The datasets presented in this article were not readily available because this data set was part of an IRB that states data cannot be shared beyond the institution and its researchers. Requests to access the datasets should be directed to dscarborough@mgh.harvard.edu.

## Ethics Statement

The studies involving human participants were reviewed and approved by Mass General Brigham Institutional Review Board. All adults participants provided written informed consent to participate in this study and all minor participants provided informed written assent with written informed consent provided by the participants' legal guardian/next of kin.

## Author Contributions

DS, EB, and SL contributed toward research design. DS and EB participated in recruitment of research participants and supervision of the project. DS and SL conducted data collection. DS, SL, and PC contributed toward data processing and analyses. All authors contributed toward data interpretation and manuscript production and revision.

## Conflict of Interest

The authors declare that the research was conducted in the absence of any commercial or financial relationships that could be construed as a potential conflict of interest.

## Publisher's Note

All claims expressed in this article are solely those of the authors and do not necessarily represent those of their affiliated organizations, or those of the publisher, the editors and the reviewers. Any product that may be evaluated in this article, or claim that may be made by its manufacturer, is not guaranteed or endorsed by the publisher.
